# Evolutionary history of arbuscular mycorrhizal fungi and genomic signatures of obligate symbiosis

**DOI:** 10.1186/s12864-024-10391-2

**Published:** 2024-05-29

**Authors:** Anna Rosling, Shadi Eshghi Sahraei, Faheema Kalsoom Khan, Alessandro Desirò, Abigail E Bryson, Stephen J Mondo, Igor V Grigoriev, Gregory Bonito, Marisol Sánchez-García

**Affiliations:** 1 Department of Ecology and Genetics, Uppsala University, Uppsala, Sweden; 2 Department of Organismal Biology, Uppsala University, Uppsala, Sweden; 3 Department of Plant Soil and Microbial Sciences, Michigan State University, East Lansing, MI USA; 4 Department of Biochemistry and Molecular Biology, Michigan State University, East Lansing, MI USA; 5 Department of Energy (DOE) Joint Genome Institute (JGI), Lawrence Berkeley National laboratory, Berkeley, CA USA; 6 Department of Plant and Microbial Biology, University of California Berkeley, Berkeley, CA 94720 USA; 7 Department of Forest Mycology and Plant Pathology, Uppsala Biocentre, Swedish University of Agricultural Sciences, Uppsala, Sweden

**Keywords:** Glomeromycota, Evolution, Phylogeny, Endogonales, Plant-fungal symbiosis

## Abstract

**Background:**

The colonization of land and the diversification of terrestrial plants is intimately linked to the evolutionary history of their symbiotic fungal partners. Extant representatives of these fungal lineages include mutualistic plant symbionts, the arbuscular mycorrhizal (AM) fungi in Glomeromycota and fine root endophytes in Endogonales (Mucoromycota), as well as fungi with saprotrophic, pathogenic and endophytic lifestyles. These fungal groups separate into three monophyletic lineages but their evolutionary relationships remain enigmatic confounding ancestral reconstructions. Their taxonomic ranks are currently fluid.

**Results:**

In this study, we recognize these three monophyletic linages as phyla, and use a balanced taxon sampling and broad taxonomic representation for phylogenomic analysis that rejects a hard polytomy and resolves Glomeromycota as sister to a clade composed of Mucoromycota and Mortierellomycota. Low copy numbers of genes associated with plant cell wall degradation could not be assigned to the transition to a plant symbiotic lifestyle but appears to be an ancestral phylogenetic signal. Both plant symbiotic lineages, Glomeromycota and Endogonales, lack numerous thiamine metabolism genes but the lack of fatty acid synthesis genes is specific to AM fungi. Many genes previously thought to be missing specifically in Glomeromycota are either missing in all analyzed phyla, or in some cases, are actually present in some of the analyzed AM fungal lineages, e.g. the high affinity phosphorus transporter Pho89.

**Conclusion:**

Based on a broad taxon sampling of fungal genomes we present a well-supported phylogeny for AM fungi and their sister lineages. We show that among these lineages, two independent evolutionary transitions to mutualistic plant symbiosis happened in a genomic background profoundly different from that known from the emergence of ectomycorrhizal fungi in Dikarya. These results call for further reevaluation of genomic signatures associated with plant symbiosis.

**Supplementary Information:**

The online version contains supplementary material available at 10.1186/s12864-024-10391-2.

## Background

Plants colonized land in the Ordovician period (ca. 475 MYA) together with associated filamentous fungi assumed based on fossil evidence representing ancestors of today’s arbuscular mycorrhizal (AM) fungi [1, 2]. The deep evolutionary origin of symbiosis between fungi and plants is further supported by the fact that the plant fungal signaling pathway that initiates AM symbiosis is ancestral to all living land plant lineages [3–6]. AM fungi are obligate mutualistic plant symbionts that cannot complete their life cycle without their plant partner. These fungi are ubiquitous in terrestrial ecosystems and have received much scientific interest because they provide nutrients (e.g. phosphorous, nitrogen) to their photosynthetic partners in exchange for fixed carbon and contribute to the overall plant fitness, during biotic (e.g. infections) or abiotic (e.g. salinity and drought) stresses [7, 8]. In the symbiosis, carbon for nutrient exchange happens in the colonized root cells where an apoplastic compartment is formed between plant and fungal membranes [9]. Here, the fungi obtain their energy and carbon in the form of sugars and lipids exuded by the plant host [10–12]. Genomic evidence for the obligate biotrophic nature of AM fungi include their loss of the ability to independently synthesize multidomain fatty acids [13] and thiamine [14]. Instead, AM fungi depend on their host for certain fatty acids as demonstrated by failed host colonization in plants with mutated fatty acids synthase genes [10, 11]. Further, the difficulties to culture AM fungi axenically has in part been attributed to their lack of a thiamine metabolic pathway [15, 16]. A set of 39 Missing Glomeromycota Core Genes (MGCGs), including fatty acids and thiamin synthesis, was proposed based on genomes from three AM fungi, *Gigaspora rosea, G. margarita* and *Rhizophagus irregularis* [16]. Re-analysis with an additional *Rhizophagus* strain detected six of the initial MGCGs among the analysed AM fungal genomes [15]. An expanded taxon sampling is expected to increase the confidence by which we can call MGCGs and determine if missing genes are uniquely missing in Glomeromycota while present in its sister lineages.

The unique role of AM fungi, as symbionts of the first land plants, has been challenged by the observation that fungi in Endogonales (Mucoromycota) also form beneficial endosymbiotic associations with early-diverging plant lineages, such as liverworts [17]. These fungi can colonize a broad range of lineages of both vascular and non-vascular plants as fine root endophytes (MFRE) and form intracellular structures resembling those of AM fungi [18, 19]. Interestingly, species within Endogonales has also been observed to form ectomycorrhizal interactions with woody plants [20, 21]. The broad host range and morphological diversity observed in Endogonales, as well as observations of dual colonization together with typical AM fungi in liverworts [22] further support the notion that MFREs may represent the earliest diverging mycobionts that facilitated land colonization by plants [18]. Symbiotic efficiency at different atmospheric carbon dioxide concentrations has led to the hypothesis that these ancestral mycorrhizal partners were later replaced by the now ubiquitous AM fungi that diversified together with flowering plants that dominate terrestrial ecosystems today [18]. AM fungi in Glomeromycota and MFREs in Endogonales (Mucoromycota) are morphologically and functionally distinct [23], and it is now well established that both form mutualistic interactions with a broad range of extant terrestrial plants [21]. The ancestor of these two mutualistic fungal lineages likely diverged before the origin of terrestrial plants [24], and ancestral reconstruction of the mycorrhizal state of land plants supports Mucoromycota as the initial symbiont [25]. However, a reconstruction where both lineages were involved cannot be ruled out, and an alternative scenario is that early land plants interacted with their common ancestor [25]. Understanding the evolutionary relationship between the two lineages and genomic signatures associated with symbiotic lifestyle can increase our understanding of the evolutionary events that shaped plant symbiotic life style in the analyzed fungal lineages.

In contrast to the endomycorrhizal symbiosis, the ectomycorrhizal (ECM) lifestyle is a mutualistic symbiosis between fungi and vascular plants that has evolved multiple times, mostly from diverse saprotrophic and endophytic ancestral fungal lineages predominantly in Dikarya but also in the Mucoromycota in the case of Endogonales [26–28]. Saprotrophic and pathogenic fungi have large repertoires of genes coding for Carbohydrate-Active enZymes (CAZyme), many of which are directly involved in the degradation of plant cell walls e.g. Plant Cell Wall Degrading Enzymes (PCWDEs) [29, 30]. Among fungal lineages in Basidiomycota and Ascomycota, the transition from saprotrophic growth to an ECM lifestyle is associated with a loss of PCWDEs, genome size expansion as a result of increased repeat content and a diversification of small secreted proteins (SSP) [26, 31, 32]. Analysis of a subset of CAZymes (45 families) indicate that PCWDEs were also lost during this independent origin of the mycorrhizal lifestyle in Endogonales [20]. However, it was noted that the numbers of CAZyme genes in Mucoromycota was generally low across species having plant-associations and saprotrophic lifestyle, and the reconstructed reduction in gene copy numbers was much smaller compared to that associated with the evolution of ECM lineages in Dikarya [20]. Similarly, a limited repertoire of genes involved in degradation of plant cell walls are detected in genomes of AM fungi [14, 33, 34] as well as in the related *Nostoc*-associated *Geosiphon pyriformis* [34]. The low CAZyme gene numbers have been attributed to the symbiotic lifestyle of AM fungi, but so far limited access to genome data has prevented a comprehensive analysis of contractions and expansions of CAZyme gene families of Glomeromycota and their sister lineages. In addition, no expansion of SSPs was observed in mycorrhizal as compared to non-mycorrhizal Mucoromycota, and together with large number of species-specific SSPs, indicates that genomic signatures of symbiotic lifestyle are different in Mucoromycota compared to Basidiomycota and Ascomycota [20].

AM fungi form a monophyletic clade but their taxonomic rank is currently fluctuating in the literature. Classified either as phylum Glomeromycota [35–37] or as the sub-phylum Glomeromycotina that together with Mortierellomycotina and Mucoromycotina, comprise the Mucoromycota [38–40]. The phylum Glomeromycota was first proposed based on early phylogenetic analysis of the rDNA genes that resolved the AM fungi as a monophyletic clade sister to Dikarya [41]. On the other hand, the phylogenomic analysis based on conserved orthologous genes, could later resolve two monophyletic clades among the paraphyletic Zygomycota [42]. With the ambition to recognize the minimum number of monophyletic phyla, the authors proposed Mucoromycota to encompass Glomeromycotina, Mucoromycotina and Mortierellomycotina, as sister to Dikarya [42]. Other authors have argued that fungi should instead be classified into monophyletic phyla that are also informative of divergence times [37]. Irrespective of taxonomic rank, phylogenomic analyses of the Kingdom Fungi resolve the three lineages as monophyletic clades based on Maximum Likelihood (ML) analysis with concatenated data and coalescence methods, but the evolutionary relationships among the three lineages remain enigmatic and a hard polytomy cannot be rejected [40]. Further, it is worth noting that the placement of Glomeromycota in the fungal tree of life remains sensitive to the evolutionary model used and filtering of fast evolving sites [43]. Morphologically, the three lineages share characters such as coenocytic hyphae and predominantly plant-based ecologies with mycorrhizal associations in two of the sister lineages [42, 44]. However, Glomeromycota stand out as a lineage of obligate symbionts of photosynthesizing partners, dominated by AM fungi that associates with plants [44] but also including *G. pyriformis* that associates with the nitrogen-fixing cyanobacteria *Nostoc* [45, 46]. Contrary to expectations, phylogenomic analysis across Glomeromycota did not place *G. pyriformis* as the sister to all other AM fungi and comparative analyses suggest that the genome signature of obligate biotrophy characteristic of the group was already present in the MRCA of the symbiotic clade [34]. The taxonomic classification of AM fungi to phylum Glomeromycota remains the most frequently used in mycorrhizal literature, and based on their unique biology we adhere to this classification and treat the three lineages as separate phyla that share a common ancestor and branch as sister to the Basidibolales, Zoopagomycota [40]. Here, Mucoromycota corresponds to Mucoromycotina [42], which encompass the mycorrhizal lineage Endogonaceae [20] and saprotrophic genera including *Mucor, Rhizopus, Umbelopsis* and others. It is relevant to note that Mortierellomycota, corresponding to Mortierellomycotina [42], have also been shown to encompass species with beneficial interactions with plants as root endophytes [47, 48]. However, these fungi are not known to form specialized structures within plant roots and to our knowledge nutrient for energy exchange has not yet been documented.

The aim of this study is to address the evolutionary relationships among extant representatives of the three fungal lineages and to analyze derived genome signature of the obligate mutualistic AM fungi. To obtain a clear picture of the early evolutionary relationships among the three phyla we carefully selected a balanced taxon sampling from all three lineages, primarily including taxa known to inhabit soil environments, in order to minimize confounding effects of adaptations to other habitats. With the selected dataset we infer the evolutionary relationships among Glomeromycota, Mortierellomycota and Mucoromycota, and examine gene family evolution for CAZymes and peptidases in order to identify coarse genomic signatures associated with the symbiotic lifestyle of Glomeromycota and Endogonales. We explore the distribution of previously identified Missing Glomeromycota Core Genes (MGCGs) across analyzed taxa and highlight interesting differences in gene content across different AM fungal lineages.

## Results

### Three evolutionary distinct lineages

**Fig. 1:**
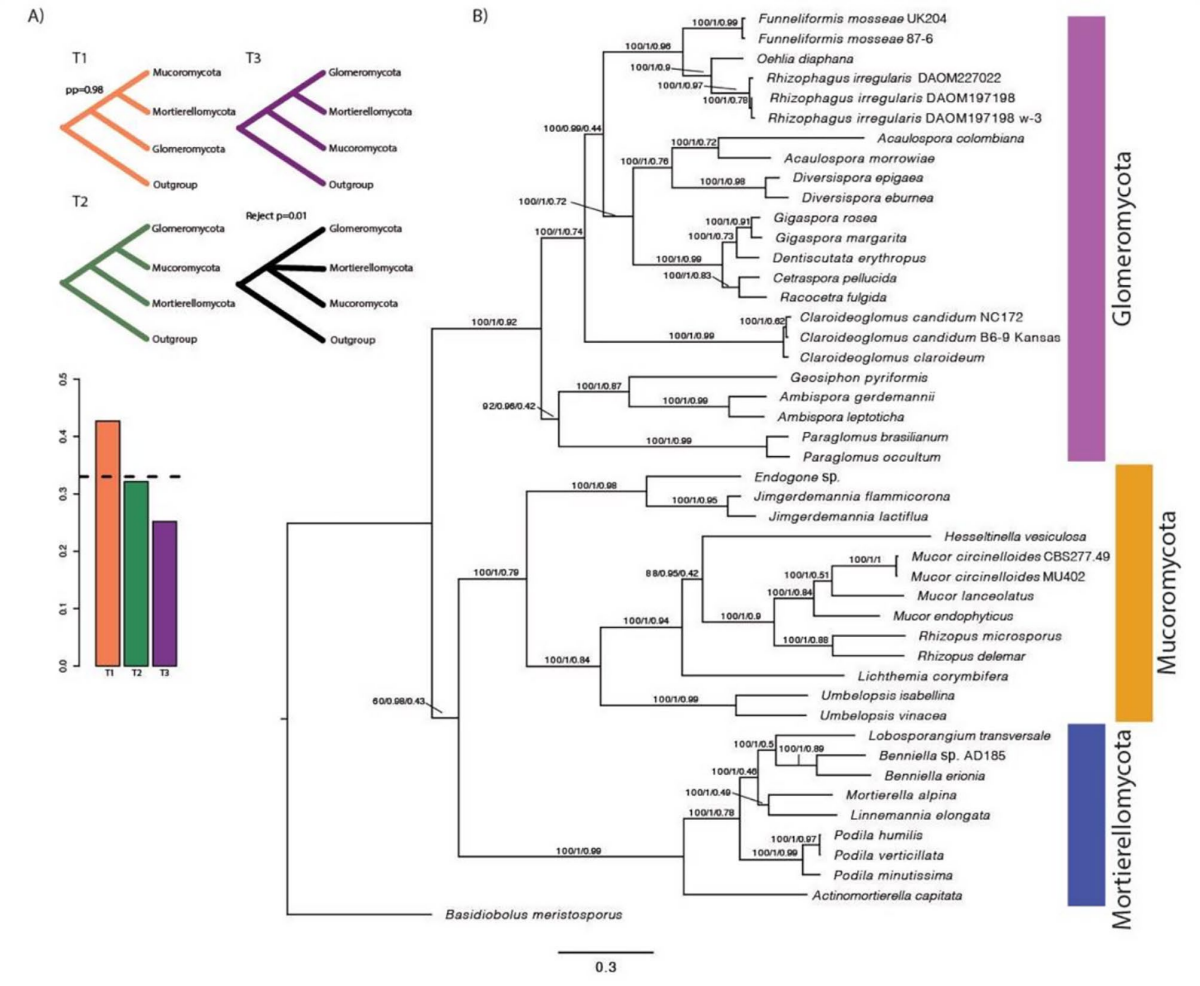
Phylogenetic relationships among the three sister phyla Glomeromycota, Mucoromycota and Mortierellomycota with *Basidiobolus meristosporus* as outgroup. (**A**) Testing support for three alternative phylogenetic relationships among the three sister phyla, based on all individual gene trees of 243 single copy orthologs (SCOs). Quartet support for the three topologies (T1-3) is summarized in the bar chart with the dashed line representing the expectation of hard polytomy. T1 corresponds to both the best ML topology and the ASTRAL topology illustrated in B. T2 and T3 correspond to alternative topologies obtained in ASTRAL. (**B**) Best maximum likelihood IQTREE phylogeny from a concatenated alignment of 243 SCOs shared at least among 50% of all forty-six taxa. Support values are shown above branches (bootstrap support from the ML analysis/local posterior probability/quartet support from the ASTRAL analysis). For details on the genomes representing taxa see Table S1

The three sister lineages are recovered as well-supported monophyletic clades with high internode supports, in both ML and coalescent-based phylogenomic analysis (Fig. 1). A polytomy scenario between the three was rejected (*p* = 0.01) and Glomeromycota is recovered as sister to the other two phyla (posterior probability = 0.98) (Fig. 1A). Within Glomeromycota, *G. pyriformis* is recovered as sister of Ambisporaceae (BS = 100%, LPP = 1, quartet support = 0.87), with Paraglomeraceae as sister of the two lineages (BS = 92%, LPP = 0.96, quartet support = 0.42) (Fig. 1B). Together these three lineages are recovered as sister to the rest of the Glomeromycota (BS = 100%, LPP = 1, quartet support = 0.92). Within Mucoromycota, Endogonales represented here by *Endogone* sp., *Jimgerdemannia flammicorona* and *J. lactiflua* branched as a sister to all other taxa in the phylum (BS = 100%, LPP = 1, quartet support = 0.79). In Mortierellomycota, *Actinomortierella capitata* separated as a sister to the rest of the taxa in the phylum (BS = 100%, LPP = 1, quartet support = 0.99) (Fig. 1B).

Compiled assembly statistics indicates some important differences between the three phyla (Table S2). Across all genomes, Glomeromycota have lower GC content, with an average of 27%, compared to 48 and 40% in Mortierellomycota and Mucoromycota respectively. However, the two assemblies representing Paraglomeraceae stand out from the other Glomeromycota with a higher GC content at 37%. Further, assembly size and predicted number of genes were higher in Glomeromycota compared to both Mortierellomycota and Mucoromycota and in both cases the estimates for Paraglomeraceae were markedly lower than the rest of the taxa in Glomeromycota (Table S2).

### Genomic signatures of biotrophy

Among 39 genes previously identified as MGCGs, only 13 could not be detected in any of the analyzed Glomeromycota, while being detected in the two sister lineages (Supplementary datafile 1). Interestingly, six of these were also not detected in the three analyzed genomes representing Endogonales, including five genes involved in thiamin metabolism (Table S3), suggesting that thiamin auxotrophy is a signature of plant biotrophy among these phyla rather than a genomic signature unique to Glomeromycota. Similarly, a gene involved in sulfonate catabolism (JLP1) was not detected in any of the mycorrhizal forming taxa analyzed. The remaining seven MGCGs were only missing in Glomeromycota, include the two fatty acid synthesis genes FAS1 and FAS2, four genes involved in detoxification and stress response, and one involved in ER quality control (Table S3). Of the 39 original MGCGs, five genes involved in sugar, alcohol and uracil metabolism and allantoin permease were missing in Glomeromycota, as well as in the analyzed genomes of Mortierellomycota and eight genes were missing in all three phyla (Table S3). Further, five of the genes previously identified as MGCGs were recovered in more than one, but not all, of the analyzed Glomeromycota genome assemblies (Figure S1). These include two genes (ARO8 and ARO9) involved in aromatic amino acid metabolism that were detected in *Paraglomus* and *Ambispora* as well as the high affinity sodium symporter PHO89 was recovered in Acaulosporaceae, Claroideoglomeraceae, Glomeraceae and *Ra. fulgida*.

**Fig. 2:**
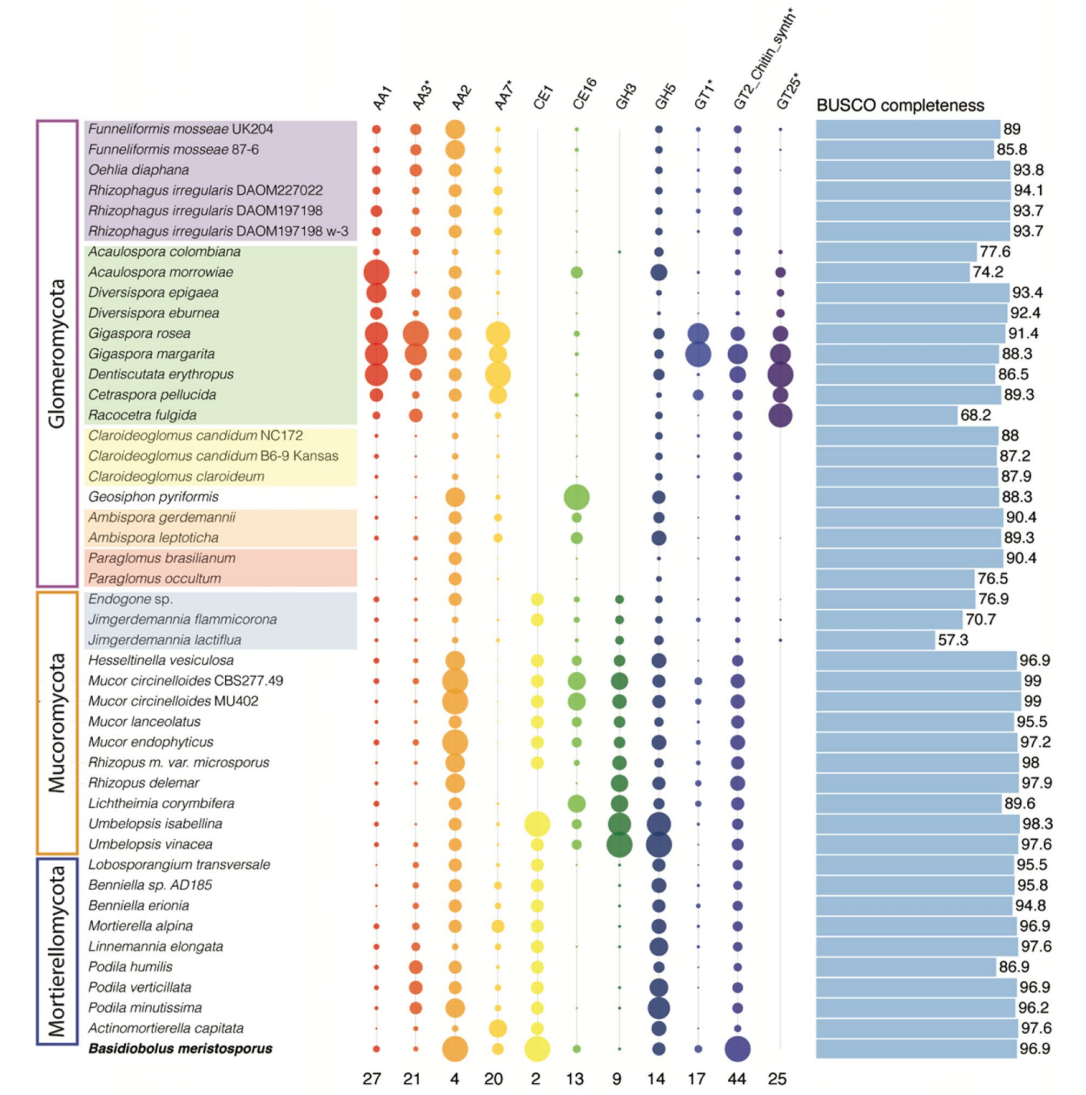
Gene copy numbers across eleven CAZyme gene families, six representing PCWDEs detected across the analyzed phyla (AA1, AA2, CE1, CE16, GH3 and GH5, Table S4) and five selected because of expansions in Gigasporaceae, here indicated with * (AA3, AA7, GT1, GT2 Chitin synthetase and GT25). Circle sizes are proportional to the number of genes annotated in each assembly, scaled individually for each gene family for readability with the maximum number of genes indicated at the bottom of each column. Estimated BUSCO completeness (Table S2) of the analyzed genome assemblies is indicated by bars to the right. Species are organized according to the phylogenetic tree in Fig. 1 and Endogonales as well as orders in Glomeromycota are highlighted by colored boxes, each color correspond to different taxonomic families

Overall, the number of predicted genes annotated as CAZymes is lower in Glomeromycota compared to Mucoromycota and Mortierellomycota (Figure S2). Among the CAZyme gene families, polysaccharide lyases stand out in the current analysis, since none were detected in any of the Glomeromycota. A handful of genes assigned to different PL families were annotated in the two sister phyla, with 2–6 genes in Mortierellomycota and 4–12 in Mucoromycota (Supplementary data 2). The gene copy number in CAZyme in the class CE are slightly more abundant in Mucoromycota and Mortierellomycota compared to Glomeromycota but numbers are overall low (Figure S2). Further our CAFE v5 (Computational Analysis of gene Family Evolution) analysis (Figure S3-S14) showed that CE4 significantly contracted in the most recent common ancestor of Glomeromycota (Figure S8). CAZymes in the class GH follow a similar pattern, interestingly, invertase (GH32) was not detected in any taxa of Glomeromycota or Mortierellomycota, nor was it detected in Mucoromycota, with the exception of the two *Umbelopsis* species that had two copies each (Supplementary data 2). The lack of GH32 genes indicates that irrespective of ecological strategy, these taxa largely lack the ability to metabolize sucrose. Numbers of enzymes with auxiliary activities (AA) on the other hand, were slightly higher in Glomeromycota compared to Mucoromycota and Mortierellomycota. Interestingly, two families in this class, AA3: involved in oxidation of alcohols or carbohydrates while simultaneously forming hydrogen peroxide [49] and AA7: involved in chitin degradation [50] were identified by the CAFE analysis as significantly expanding within specific lineages of Glomeromycota (Figures S5-S6). Similarly, two families of glycoside transferase (GT1 and GT2-chitin synthase [51] significantly expanded in Gigasporaceae (Fig. 2, S12, S13). While most of the Glomeromycota have lower gene counts for the class GT, the Gigasporaceae have gene counts similar to the other two phyla. Overall, all three phyla have low gene copy numbers for CBM (Figure S2).

Based on our CAFE analysis, we detected one expansion and 39 contractions in CAZyme gene families at the branch leading to the Glomeromycota (Figure S3). We also detected 15 expansions of CAZyme gene families and 17 contractions at the branch leading up to the Mortierellomycota. In Mucoromycota we detected four expansions and three contractions of CAZyme gene families. However, in the branch leading to Endogonales we detected two expansions and 27 contractions in CAZyme gene families (Figure S3). In our dataset, eleven CAZyme gene families showed significant changes in number (Figures S4-S14), but only three had significant changes at the nodes discussed above, for example AA7 and AA11 significantly contracted on the branch leading to Mucoromycota, and CE4 and AA11 significantly contracted in the most recent common ancestor of the three phyla (Mucoromycota, Mortierellomycota and Glomeromycota) (Figure S6-S8).

Out of the 17 CAZyme gene families classified as PCWDEs and previously analyzed in relation to biotrophic lifestyle switches [14, 20, 26, 32], eleven were recorded at very low numbers in a handful of genomes and were not recorded at all in the outgroup (Table S4). Thus, the absence of these gene families, including typical PCWDEs such as GH6, GH7 and CBM1, in genomes from Glomeromycota cannot be interpreted as an evolving signature of their obligate biotrophic lifestyle, since they were not present in the most recent common ancestor of these phyla. Of the remaining six gene families, CE1 and GH3 are absent or near absent in Glomeromycota, GH5 is recorded at somewhat lower numbers in Glomeromycota compared to the other phyla while AA1, which includes laccases, and peroxidases in AA2 and acetyl esterase in CE16 are recovered in all analyzed genomes (Fig. 2). AA1 is significantly expanded in the ancestor of Diversisporales and Glomerales, and further expanded in Diversisporales (Figure S4).

We identified twelve CAZyme gene families putatively missing in Glomeromycota while found in the other two phyla (Table S5). These include two families of chitin binding modules (CBM5 and 12), eight glycoside hydrolase families and two polysaccharide lysase families (PL14 and 36). Most of these gene families are small and rare across all phyla but the bacterial glycoside hydrolase GH8 and PL14 may deserve further analysis that may reveal if these are novel genes uniquely missing in Glomeromycota (Table S5).

Overall, the copy numbers in peptidases gene families as identified based on the MEROPS database, were similar across all three phyla (Figure S15). Among the families of proteolytic enzymes, the asparagine peptide lyases (APL) gene family stands out with no genes detected in Mucoromycota and Mortierellomycota (Figure S15). In Glomeromycota on the other hand, APL (N9) was detected in all taxa except *Ac. colombiana*, *Diversispora epigaea* and *D. eburnea*. In *R. irregularis* (A1) APL (N6) was found and APL (N11) was detected in *G. pyriformis*. On the branch leading to Glomeromycota we detected four expansions and 35 contractions. On the branch leading to Mortierellomycota we identified 22 expansions and nine contractions, while on the branch leading to Mucoromycota we identified three expansions and six contractions (Figure S16). For Endogonales, we detected nine expansions and 24 contractions of peptidase gene families (Figure S16). In the most recent common ancestor of the three phyla, we observed 27 contracting peptidases families and no expansions (Figure S16). Based on the CAFE analysis, 16 peptidases show significant changes in number, three of them have significantly expanded on the branch leading to Mortierellomycota (M13, S09X and S12; Figures S17-19) and five show significant expansions on the branch leading to Gigasporaceae (S09X, A01A, C19, S09C and S10; Figures S18, S20-S23).

## Discussion

In this paper, we use broad taxon sampling to analyze the evolutionary relationships of Glomeromycota, Mucoromycota and Mortierellomycota, which together represent a monophyletic lineage whose relationships have remained unresolved despite increasing genomic resources [40]. Importantly, recent phylogenomic analysis support Basidiobolales, Zoopagomycota as the sister to these three clades [39, 40], revising the earlier placement of the three as sister to Dikarya [38]. Using a balanced taxon sampling with broad taxonomic representation of recently released genome assemblies of all three lineages and with *B. meristosporus* as the outgroup, we were able to reject a hard polytomy and present a phylogenetic analysis supporting Glomeromycota as sister to a clade composed of Mortierellomycota and Mucoromycota (Fig. 1). This relationship has not previously been supported in phylogenomic analyses including taxa of Glomeromycota, possibly due to the limited representation of Mortierellomycota and Mucoromycota taxa in those analyses [34, 40, 52]. In line with earlier studies, our phylogenomic analyses support that the symbiotic fungi in Glomeromycota and Endogonales (Mucoromycota) represent two independent origins of mutualistic plant symbiotic lifestyles. The alternative scenario that the two lineages represent one evolutionary event appears less likely given their functional and morphological differences [23].

The recovered relationships within Glomeromycota are well supported and in line with earlier findings [52]. Among these obligate plant symbionts, *G*. *pyriformis* stands out by forming a symbiotic interaction with *Nostoc* cyanobacteria instead of plants. Nevertheless, the interaction does not appear to represent an ancestral state as revealed by phylogenomic analysis [34]. With our broad taxon sampling, *G*. *pyriformis* was recovered as a well-supported sister to members of the Ambisporaceae (BS = 100%, LPP = 1, quartet support = 0.87) (Fig. 1). This topology differs from a previous analysis in which *G. pyriformis* was recovered as sister to Ambisporaceae and Paraglomeraceae [34]. In a later analysis, however, *G*. *pyriformis* was recovered as sister to *Ambispora leptoticha* and separate from *Paraglomus occultum*, which was recovered as sister to other AM fungi [34]. In our analysis on the other hand, Paraglomeraceae is recovered as sister to *G*. *pyriformis* and Ambisporaceae, and together the three lineages are sister to all other AM fungi. However, these relationships are not strongly supported (BS = 92%, LPP = 0.96, quartet support = 0.42) (Fig. 1). There are several reasons for why the recovered topologies differ among studies, including taxon sampling, strategies for selecting orthologs, handling of missing data and phylogenetic inference methods used [53–55].

Our summary of assembly statistics supports previous observations of high GC content, smaller assembly size and lower number of genes in Paraglomeraceae compared to other Glomeromycota [34]. These genome characteristics make Paraglomeraceae more similar to taxa in the other phyla analyzed in this study. To resolve the phylogenetic relationships among early divergent lineages of Glomeromycota, future studies should include taxa from currently missing lineages, such as the Archaeosporaceae [56], and should carefully analyze the effect of selected gene sets on phylogenetic reconstructions [55].

While Glomeromycota exclusively encompass obligate biotrophic AM fungi and *G. pyriformis*, the other two phyla represent different ecological lifestyles including facultative mutualistic symbionts, endophytes, saprotrophs and opportunistic pathogens [20, 38]. In Dikarya, switching to a mycorrhizal habit has occurred as multiple independent evolutionary events and is known to be associated with genomic signatures such as contractions in CAZyme gene families, in particular those associated with plant cell wall degradation [27]. Hence, the absence of many typical PCWDEs in analyses of AM fungal genomes has been interpreted as a functional gene signature of obligate biotrophic lifestyle [14–16]. While we see contractions of CAZymes in the most recent common ancestor of Glomeromycota (Figure S3), these do not correspond to PCWDEs with the exception of AA1 (Figure S4), that shows significant contractions. Instead, we demonstrate that most of the PCWDEs gene families are also absent in the sister phyla, as well as in the outgroup. Thus, we suggest that the low numbers of PCWDEs in AM fungi is not an adaptation to their obligate plant symbiotic lifestyle, rather it represents an ancestral phylogenetic signature. Ten different families of PCWDEs were detected in Endogonales (Table S4), all of them in low copy number. PCWDEs abundantly detected among wood decay Basidiomycetes likely diversified early in the evolutionary history of Dikarya [57], when the three phyla studied here had already diverged and were evolving as separate lineages [24]. An additional interesting finding is the number of copies of GT25 in members of Diversisporales (Fig. 2), this CAZyme family was not present at the root of our tree, therefore an analysis with an expanded outgroup that includes members of Dikarya will provide insights into the origin and evolution of this family.

Other genomic signatures of symbiotic lifestyle are likely to characterize mycorrhizal fungi in these lineages. For instance, in line with earlier studies [34, 58–60], fatty acid synthase homologous genes were not detected in any of the analyzed Glomeromycota genomes. This further supports the notion that the obligate nature of the AM fungal symbiosis is maintained through the provision of fatty acids as a source of energy and carbon from the host [10–12]. Other core genes previously suggested to be missing only in Glomeromycota, so called MGCGs, were either found to be missing in all analyzed phyla, or were actually present in some Glomeromycota genomes (Table S3, Figure S1). With the increase in available genome assemblies from a broad range of AM fungal taxa [52], it would be timely to repeat the comprehensive analysis of the genetic basis for auxotrophy among AM fungi previously performed on only a handful of taxa [15]. However, such efforts were outside the scope of the current study.

## Conclusion

Based on a broad taxon sampling we present a well-supported phylogeny with Glomeromycota, including all AM fungi, as sister to a clade comprising Mucoromycota and Mortierellomycota. We find that in these lineages the evolutionary transitions to mutualistic plant symbiosis in AM fungi and symbiotic MFRE Endogonales happened in a genomic background profoundly different from that known from the emergence of ectomycorrhizal fungi in Dikarya. Specifically, losses of typical PCWDEs cannot be attributed to the mycorrhizal symbioses, since they were not inferred to be present in the ancestor of the three phyla. With the expanded taxon sampling we found that many genes previously thought to be missing in all AM fungi are either present in some of them or missing also in their sister lineages. These results call for further reevaluation of genomic signatures associated with plant symbiosis among early diverging fungi.

## Materials and methods

### Taxon sampling, gene prediction and functional annotation

In our analysis, Glomeromycota is represented by 23 genome assemblies of 15 species across eight families (Table S1). We included 17 genome assemblies with BUSCO completeness estimates of at least 82% from AM fungal genomes assembled from combined reads from multiple separately amplified and sequenced nuclei [52, 61]. Further, we retrieved genome data from the Joint Genome Institute (JGI) and GenBank, six from Glomeromycota [34, 58, 59, 62], 13 from Mucoromycota [20, 63–69] and nine from Mortierellomycota, six published [47, 63, 70] and three unpublished. The genome of *Basidiobolus merisporus* (Zoopagomycota) [63] was included as an outgroup based on phylogenetic placement in a recent analysis of the fungal tree of life [40] (Table S1). Species names are updated following taxonomic revisions [71].

For genomes published by Montoliu-Nerin and co-workers [52, 61], gene prediction was performed using an in-house annotation pipeline (v4.0) (https://bitbucket.org/scilifelab-lts/genemark_fungal_annotation/src/master/). In brief, RepeatModeler (v1.0.8) [72] was used to predict repeats and transposable elements from each assembly. The RepeatModeler library was thereafter filtered to obtain protein-coding genes before using RepeatMasker (v4.0.7) [72] to mask each genome assembly. MAKER (V3.01.1-beta) [73] was then used to align Uniprot/Swiss-Prot databases [74, 75] to the repeat masked genome assemblies. Thereafter, GeneMark-Es (4.33-es_perl5.24.1) [76] was used to predict protein coding genes from repeat mask genome assemblies which provided genome location of Uniprot/Swiss-Prot proteins. In this step, a minimum contig size of 10 Mb from each assembly was included in the training database of GeneMark-Es. For the remaining genomes, we used predicted genes from the original sources (Table S1-S2). To ensure consistent annotation for downstream analysis, all predicted genes were annotated with Funannotate v.1.8.9 [77], using the following databases: Swiss-Prot [78], InterPro [79], pfam (ref), eggnog [80], MEROPS, the peptide database, v.12 [81] and databases of automated CAZyme annotation (dbCAN) v.9 [82].

### Phylogenetic analysis and topology testing

Single copy orthologs (SCOs) of predicted genes were identified with Orthofinder v.2.4.0 [83] using default parameters. The identified SCOs present in at least 50% of the taxa were used for phylogenetic inference. Amino acid sequences were aligned with MAFFT v.7.407 using the --auto setting [84, 85]. Poorly aligned regions were removed with trimAl v.1.4.1 [86] with a gap threshold (-gt) of 0.2. For maximum likelihood (ML) phylogenetic analysis, the individual alignments were concatenated into a supermatrix with the geneSticher.py script [87]. A phylogenetic inference based on the concatenated dataset was made with IQ-TREE v.2.0 [88] with 100 bootstrap replicates. The best-fit model for each gene partition and the best-fit partition scheme were estimated with ModelFinder [89]. A second phylogenetic inference consistent with the coalescent species model was performed using ASTRAL III v.5.7.3 [90]. We inferred individual gene trees with IQ-TREE using the automated detection for best-fit model (MFP). The topological robustness was assessed with local posterior probabilities (LPP) and quartet supports. We evaluated the support for the three possible relationships among Glomeromycota, Mucoromycota and Mortierellomycota, based on the support among individual gene trees for alternative branching orders and performed a polytomy test with ASTRAL-III v.5.7.3 [91].

### Gene content analysis

To test for differences in gene content among Glomeromycota, Mucoromycota and Mortierellomycota, we used three datasets, CAZyme, peptidases and Pfam domains, generated from the functional annotation in Funannotate. The CAZymes include five classes of enzymes that metabolize carbohydrates and glycoconjugates organized into the glycoside hydrolases (GHs), glycosyl transferases (GTs), polysaccharide lyases (PLs), carbohydrate esterases (CEs) and auxiliary activities (AAs) as well as genes with carbohydrate-binding modules (CBMs) while the peptidases include eight MEROPS families of proteolytic enzymes with different starting points for the catalysis of peptides. Manual inspection of the CAZyme and peptidases summary tables with total number of genes per family and genome assembly drew our attention to two cases, firstly one gene belonging to the CAZyme polysaccharide lyase gene class (PL3) and one belonging to the auxiliary activities gene family AA9 was annotated in only *Acaulospora colombiana* of the Glomeromycota and two gene copies in the MEROPS Asparagine peptide lyase family (NO9) was annotated in only one genome assembly outside of Glomeromycota. To verify these annotations, the three genes were extracted from *A. colombiana* and the AA9 genes from the *Podila verticillata* genome assemblies and blasted against the NCBI database using both the highly similar and somewhat similar settings. Subsequently, all genes with these annotations were extracted from the dataset and separately aligned. Complete genes could not be confirmed by targeted BLASTn search and alignment, we thus conclude that it is possible but unlikely that one gene in PL3 and AA9 is present in a single Glomeromycota genome. Similarly, the asparagine peptide lyase gene (NO9) from *P. verticillata* could not be confirmed. We concluded that the annotation was not reliable and removed the scored presence of the gene from the output file prior to downstream analysis. The gene family copy numbers for CAZymes and peptidases were visualized across phyla.

In order to determine the distribution and abundance of 39 genes previously identified as MGCGs [16], we performed a BLASTp search on all analyzed genomes assemblies using the *Sacharomyces cerevisiae* reference sequences for the MGCGs as query. Similar to earlier studies [15, 16], all BLAST hits with an e-value < 10^− 5^ were evaluated and genes were counted as present in a genome using a bitscore cut off at 100. These thresholds captured distinct similarity gaps across the hits for all genes (Supplementary data 1). Detection of MGCGs across the three phyla was based on average number of gene copies per genome assembly and number of assemblies with gene copies.

To identify nodes of significant changes in gene family sizes (so called rapidly evolving gene families), gene family evolution analysis in the CAZyme and peptidase datasets were computed using CAFE (Computational Analysis of gene Family Evolution) analysis (v5) [92] with a p-value cutoff of 0.05. Prior to this, the phylogeny inferred from the ML analyses was converted into an ultrametric tree using r8s (v1.81) [93]. The optimal smoothing parameter was obtained by cross-validation analysis. Divergence times were estimated using a Penalized Likelihood method, Truncated Newton algorithm, smoothing parameter value of 2.50 and three calibration points for Glomeromycota (407 MYA) based on the fossil *Glomites rhyniensis*, Mucorales (315 MYA) based on the fossil *Protoascon missouriensis* and Endogonaceae (247 MYA) based on the fossil *Jimwhitea circumtecta* [94]. CAZymes classified as PCWDEs have been found to be completely or partially lost in lineages of Basidiomycota ECM fungi [26] and similar patterns are observed for ECM fungi in Ascomycota [32]. We specifically examined the copy number of 17 CAZyme gene families classified as PCWDEs in earlier studies of different mycorrhizal lineages [14, 20, 26, 32] (Table S4).

### Electronic supplementary material

Below is the link to the electronic supplementary material.

Supplementary Material 1

Supplementary Material 2

Supplementary Material 3

## Data Availability

Published genome datasets analyzed in the current study are available in the public repositories listed in the associated publications for details see Table S1. Unpublished genome datasets are available in the JGI MycoCosm fungal genome resource (https://mycocosm.jgi.doe.gov/MorAD185_1_1/MorAD185_1_1.home.html; https://mycocosm.jgi.doe.gov/Moralp1_1/Moralp1_1.home.html and https://mycocosm.jgi.doe.gov/Morcap1/Morcap1.home.html). FunnAnotate output files as well as alignment and tree files are available in the associated FigShare project 10.17044/scilifelab.23553426.

## Abbreviations

Aas: auxiliary activities
AM: arbuscular mycorrhiza
BS: bootstrap support
CAFÉ: Computational Analysis of gene Family Evolution
CAZymes: Carbohydrate–active Enzymes
CBMs: carbohydrate–binding modules
Ces: carbohydrate esterases
ECM: ectomycorrhiza
GHs: glycoside hydrolases
GTs: glycosyl transferases
LPP: local posterior probability
MFRE: Mucoromycota fine root endophytes
MGCG: Missing Glomeromycota Core Gene
ML: Maximum likelihood
nMDS: Non–metric multidimensional
PCWDE: Plant Cell Wall Degrading Enzyme
PLs: polysaccharide lyases
SCOs: single copy orthologs
