# Supplementary material for: Evolutionary history of arbuscular mycorrhizal fungi and genomic signatures of obligate symbiosis
Source: BMC Genomics. 2024 May 29;25:529. doi: 10.1186/s12864-024-10391-2 (PMC11134847; doi:10.1186/s12864-024-10391-2)
Supplement: Supplementary file 3 — Supplementary Material 3 [file 12864_2024_10391_MOESM3_ESM.docx]

**Evolutionary history of arbuscular mycorrhizal fungi and genomic signatures of obligate symbiosis**

Anna Rosling^1*, **^, Shadi Eshghi Sahraei^1*^, Faheema Kalsoom Khan^2^, Alessandro Desirò^3^, Abigail E Bryson^3^, Stephen J Mondo^4^, Igor V Grigoriev^4,5^, Gregory Bonito^3^, Marisol Sánchez-García^1,6 **^

**Supplementary tables and figures**

**Table S1.** List of published genome assemblies included in the analysis.

**Table S2.** Genome statistics for all taxa included in the analysis.

**Table S3.** Genes identified as Missing Glomeromycota Core Genes (MGCGs)

**Table S4.** CAZyme genes families previously analyzed as plant cell wall degrading enzymes (PCWDEs)

**Table S5.** CAZyme genes families identified in this study as missing only in Glomeromycota.

**Figure S1.** Gene copy number for five genes previously identified as MGCGs

**Figure S2.** Total number of genes annotated into the six CAZyme classes

**Figure S3.** Expansions and contractions of carbohydrate-active enzyme (CAZyme) gene families

**Figure S4.** Expansions and contractions of CAZyme family AA1

**Figure S5.** Expansions and contractions of CAZyme family AA3

**Figure S6.** Expansions and contractions of CAZyme family AA7

**Figure S7.** Expansions and contractions of CAZyme family AA11

**Figure S8.** Expansions and contractions of CAZyme family CE4

**Figure S9.** Expansions and contractions of CAZyme family CE10

**Figure S10.** Expansions and contractions of CAZyme family GH16

**Figure S11.** Expansions and contractions of CAZyme family GH47

**Figure S12.** Expansions and contractions of CAZyme family GT1

**Figure S13.** Expansions and contractions of CAZyme family GT2 chitin synthase

**Figure S14.** Expansions and contractions of CAZyme family GT10

**Figure S15.** Total number of genes annotated into the eight families of proteolytic enzymes

**Figure S16.** Expansions and contractions of Peptidase gene families

**Figure S17.** Expansions and contractions of peptidase family M13

**Figure S18.** Expansions and contractions of peptidase family S09X

**Figure S19.** Expansions and contractions of peptidase family S12

**Figure S20.** Expansions and contractions of peptidase family A01A

**Figure S21.** Expansions and contractions of peptidase family C19

**Figure S22.** Expansions and contractions of peptidase family S09C

**Figure S23.** Expansions and contractions of peptidase family S10

**Supplementary data file 1.** Missing Glomeromycota Core Genes (MGCG) as identified by Tang et al 2016, presence across Glomeromycota, Mucoromycota and Mortierellomycota scored by BLASTp.

**Supplementary data file 2.** Carbohydrate-active enzyme (CAZyme) gene families in Glomeromycota, Mucoromycota and Mortierellomycota.

**Table S1**. List of published genome assemblies included in the analysis.

| Number Id | Species | Strain Id | Phyla | Published in |
| --- | --- | --- | --- | --- |
| 1 | *Funneliformis mosseae* * | UK204 | Glomeromycota | Montoliu-Nerin et al., 2021 (49) |
| 2 | *Funneliformis mosseae* | 87-6 pot B 2015 | Glomeromycota | Montoliu-Nerin et al., 2021 (49) |
| 3 | *Oehlia diaphana *** | DAOM 227022 V1.0 | Glomeromycota | Morin et al., 2019 (54) |
| 4 | *Rhizophagus irregularis **** | DAOM229456 | Glomeromycota | Morin et al., 2019 (54) |
| 5 | *Rhizophagus irregularis* | DAOM197198 | Glomeromycota | Chen et al., 2018 (60) |
| 6 | *Rhizophagus irregularis-*w3 | DAOM197198 | Glomeromycota | Montoliu-Nerin et al., 2021 (49) |
| 7 | *Acaulospora colombiana* | CL356 | Glomeromycota | Montoliu-Nerin et al., 2021 (49) |
| 8 | *Acaulospora morrowiae* | CL551 | Glomeromycota | Montoliu-Nerin et al., 2020 (59) |
| 9 | *Diversispora epigaea* | IT104 | Glomeromycota | Sun et al., 2019 (55) |
| 10 | *Diversispora eburnea* | AZ414A | Glomeromycota | Montoliu-Nerin et al., 2021 (49) |
| 11 | ***Gigaspora rosea*** | **DAOM 194757** | Glomeromycota | Morin et al., 2019 (54) |
| 12 | *Gigaspora margarita* | 120-4 pot B 10/14 | Glomeromycota | Montoliu-Nerin et al., 2021 (49) |
| 13 | *Dentiscutata erythropus* | MA453B | Glomeromycota | Montoliu-Nerin et al., 2021 (49) |
| 14 | *Cetraspora pellucida* | FL966 | Glomeromycota | Montoliu-Nerin et al., 2021 (49) |
| 15 | *Racocetra fulgida* | IN212 | Glomeromycota | Montoliu-Nerin et al., 2021 (49) |
| 16 | *Claroideoglomus candidum* | NC172 | Glomeromycota | Montoliu-Nerin et al., 2021 (49) |
| 17 | *Claroideoglomus candidum* | (CCK) pot B 6-9 | Glomeromycota | Montoliu-Nerin et al., 2021 (49) |
| 18 | *Claroideoglomus claroideum* | SA101 | Glomeromycota | Montoliu-Nerin et al., 2020 (59) |
| 19 | *Geosiphon pyriformis* | - | Glomeromycota | Malar et al., 2021 (30) |
| 20 | *Ambispora gerdemannii* | MT106 | Glomeromycota | Montoliu-Nerin et al., 2021 (49) |
| 21 | *Ambispora leptoticha* | FL130A | Glomeromycota | Montoliu-Nerin et al., 2021 (49) |
| 22 | *Paraglomus brasilianum* | BR232B | Glomeromycota | Montoliu-Nerin et al., 2021 (49) |
| 23 | *Paraglomus occultum* | IA702 | Glomeromycota | Montoliu-Nerin et al., 2021 (49) |
| 24 | *Endogone sp.* | FLAS 59071 | Mucoromycota | Chang et al., 2019 (28) |
| 25 | *Jimgerdemannia flammicorona* | AD002 | Mucoromycota | Chang et al., 2019 (28) |
| 26 | *Jimgerdemannia lactiflua* | OSC166217 | Mucoromycota | Chang et al., 2019 (28) |
| 27 | *Hesseltinella vesiculosa* | NRRL3301 V2.0 | Mucoromycota | Mondo et al., 2017 (62) |
| 28 | *Mucor circinelloides* | CBS277.49 | Mucoromycota | Corrochano et al., 2016 (63) |
| 29 | *Mucor circinelloides* | MU402 | Mucoromycota | Navarro-Mendoza et al., 2019 (66) |
| 30 | *Mucor lanceolatus* | UBOCC-A-109153 | Mucoromycota | Lebreton et al., 2020 (57) |
| 31 | *Mucor endophyticus* | UBOCC-A-113049 | Mucoromycota | Lebreton et al., 2020 (57) |
| 32 | *Rhizopus microsporus var. Microsporus* | ATCC52813 | Mucoromycota | Mondo et al., 2017 (62) |
| 33 | *Rhizopus delemar* | RA99 880 | Mucoromycota | Ma et al., 2009 (58) |
| 34 | *Lichtheimia corymbifera* | JMRC:FSU:9682 | Mucoromycota | Schwartze et al., 2014 (54) |
| 35 | *Umbelopsis isabellina* | WA0000067209 | Mucoromycota | Muszewska et al., 2021 (65) |
| 36 | *Umbelopsis vinacea* | WA0000051536 | Mucoromycota | Muszewska et al., 2021 (65) |
| 37 | *Lobosporangium transversale* | NRRL 3116 | Mortierellomycota | Mondo et al., 2017 (62) |
| 38 | *Mortierella sp_AD* | AD185 | Mortierellomycota | Unpublished genome ^1^ |
| 39 | *Benniella erionia* | GBAus27b | Mortierellomycota | Vandepol et al., 2021 (43) |
| 40 | *Mortierella alpina* | AD266 | Mortierellomycota | Unpublished genome ^2^ |
| 41 | *Linnemannia elongata* | AG-77 v.2 | Mortierellomycota | Uehling et al., 2017 (67) |
| 42 | *Podila humilis* | PM1414 | Mortierellomycota | Vandepol et al., 2021 (43) |
| 43 | *Podilia verticillate***** | NRRL6337 | Mortierellomycota | Vandepol et al., 2021 (43) |
| 44 | *Podila minutissima* | AD051 | Mortierellomycota | Vandepol et al., 2021 (43) |
| 45 | *Actinomortierella capitata* | AV005 | Mortierellomycota | Unpublished genome ^3^ |
| - | *Basidiobolus meristosporus* | CBS 931.73 v1.0 | *Outgroup* | Mondo et al., 2017 (62) |

* The strain UK2014 was initially listed as *F. caledonius* but has been reidentified as *F. mosseae* (W. Wheeler personal communication) in line with phylogenomic analyses (49).

** The genus *Oehlia* is represented by *O. diaphana* strain DAOM227022. A genome assembly of this strain was previously included in phylogenomic analyses under the name *R. cerebriforme* (30, 54, 59), but based on molecular and morphological data, this strain was shown to represent *O. diaphana* (68).

*** DAOM229456 corresponds to MUCL43196 in (54) where this strain was published under the name *R. diaphanus but* phylogenetic analyses suggest that this strain actually belongs to *R. irregularis* (68).

**** Species name kept in this paper as previously earlier published, although the species name should probably be *P. humilis*.

1. <https://mycocosm.jgi.doe.gov/MorAD185_1_1/MorAD185_1_1.home.html>
2. <https://mycocosm.jgi.doe.gov/Moralp1_1/Moralp1_1.home.html>
3. <https://mycocosm.jgi.doe.gov/Morcap1/Morcap1.home.html>

**Table S2.** Genome statistics for all taxa included in the analysis.

| **Species name** | **Strain number** | **Assembly size (Mb)** | **Number of contigs** | **N50 (bp)** | **%GC** | **Number of genes** | **BUSCO ^#^** |
| --- | --- | --- | --- | --- | --- | --- | --- |
| *Funneliformis mosseae* * | UK204 | 146 | 31,052 | 9,933 | 26 | 20,376 | C: 251  F: 28 |
| *Funneliformis mosseae* | 87-6 pot B 2015 | 146 | 27,134 | 11,988 | 25 | 19,051 | C: 250  F: 31 |
| *Oehlia diaphana* ** | DAOM227022 | 137 | 2,592 | 147,873 | 22 | 21,549 | C: 278  F: 7 |
| *Rhizophagus irregularis* | DAOM197198 | 126 | 2,764 | 137,499 | 24 | 23,252 | C: 283  F: 2 |
| *Rhizophagus irregularis* | DAOM197198 (w3) | 125 | 11,196 | 51,491 | 26 | 26,580 | C: 271  F: 13 |
| *Rhizophagus irregularis* *** | DAOM229456 | 116 | 15,939 | 21,163 | 27 | 23,258 | C: 281  F: 5 |
| *Acaulospora colombiana* | CL356 | 300 | 75,131 | 8,208 | 29 | 13,269 | C: 110  F: 103 |
| *Acaulospora morrowiae* | CL551 | 219 | 64,585 | 6,189 | 28 | 21,890 | C: 235  F: 47 |
| *Diversispora epigaea* | IT104 | 157 | 731 | 452,614 | 25 | 28,348 | C: 278  F: 7 |
| *Diversispora eburnea* | AZ3414A | 65 | 9,803 | 22,612 | 26 | 13,318 | C: 237  F: 45 |
| ***Gigaspora rosea*** | **DAOM 194757** | 568 | 7,499 | 232,087 | 27 | 31,331 | C: 278  F: 6 |
| *Gigaspora margarita* | 120-4 potB 10/4 | 575 | 173,647 | 5,875 | 27 | 57,019 | C: 249  F: 32 |
| *Dentiscutata erythropus* | MA453B | 294 | 73,964 | 7,545 | 28 | 34,459 | C: 253  F: 25 |
| *Cetraspora pellucida* | FL966 | 435 | 90,930 | 10,017 | 26 | 26,560 | C: 251  F: 27 |
| *Racocetra fulgida* | IN212 | 296 | 105,307 | 4,839 | 28 | 24,442 | C: 101  F: 94 |
| *Claroideoglomus candidum* | NC172 | 68 | 12,232 | 15,216 | 28 | 16,486 | C: 246  F: 33 |
| *Claroideoglomus candidum* | (CCK) pot B 6-9 | 70 | 12,603 | 15,877 | 28 | 16,750 | C: 249  F: 32 |
| *Claroideoglomus claroideum* | SA101 | 68 | 11,246 | 15,947 | 28 | 16,816 | C: 239  F: 43 |
| *Geosiphon pyriformis* | - | 128 | 792 | 703,035 | 28 | 11,949 | C: 271  F: 8 |
| *Ambispora gerdemannii* | MT106 | 88 | 19,363 | 10,266 | 28 | 15,654 | C: 264  F: 20 |
| *Ambispora leptoticha* | FL130A | 197 | 58,506 | 5,886 | 24 | 16,806 | C: 253  F: 30 |
| *Paraglomus brasilianum* | BR232B | 58 | 7,115 | 21,894 | 37 | 12,781 | C: 263  F: 22 |
| *Paraglomus occultum* | IA702 | 50 | 8,053 | 16,033 | 37 | 12,217 | C: 224  F: 38 |
| *Endogone sp.* | FLAS59071 | 96 | 15,409 | 11,261 | 39 | 9,569 | C: 175  F: 43 |
| *Jimgerdemannia flammicorona* | AD002 | 231 | 35,354 | 10,860 | 43 | 13,838 | C: 168  F: 53 |
| *Jimgerdemannia lactiflua* | OSC166217 | 180 | 45,525 | 5,625 | 44 | 12,651 | C: 111  F: 90 |
| *Hesseltinella vesiculosa* | NRRL3301 V2.0 | 27 | 114 | 571,097 | 46 | 11,141 | C: 281  F: 7 |
| *Mucor circinelloides* | CBS277.49 | 37 | 26 | 4,318,338 | 42 | 11,686 | C: 270  F: 12 |
| *Mucor circinelloides* | MU402 | 37 | 24 | 4,574,267 | 42 | 11,843 | C: 282  F: 2 |
| *Mucor lanceolatus* | UBOCC-A-109153 | 43 | 1,531 | 141,562 | 34 | 10,644 | C: 279  F: 7 |
| *Mucor endophyticus* | UBOCC-A-113049 | 35 | 159 | 1,956,696 | 34 | 11,445 | C: 285  F: 4 |
| *Rhizopus microsporus* | ATCC52813 | 26 | 131 | 1,118,338 | 37 | 10,905 | C: 283  F: 5 |
| *Rhizopus delemar* | RA99 880 | 46 | 81 | 3,104,119 | 35 | 17,467 | C: 242  F: 35 |
| *Lichtheimia corymbifera* | JMRC:FSU:9682 | 34 | 207 | 367,562 | 40 | 13,404 | C: 262  F: 6 |
| *Umbelopsis isabellina* | WA0000067209 | 22 | 60 | 1,335,078 | 42 | 9,033 | C: 276  F: 8 |
| *Umbelopsis vinacea* | WA0000051536 | 23 | 148 | 1,328,092 | 43 | 9,240 | C: 276  F: 11 |
| *Lobosporangium transversale* | NRRL 3116 | 43 | 138 | 672,590 | 42 | 11,822 | C: 282  F: 6 |
| *Mortierella sp_AD* | AD185 | 56 | 51 | 2,641,002 | 50 | 15,422 | C: 287  F: 3 |
| *Benniella erionia* | GBAus27b | 45 | 140 | 820,600 | 51 | 13,953 | C: 283  F: 4 |
| *Mortierella alpina* | AD266 | 40 | 40 | 2,137,591 | 50 | 12,145 | C: 288  F: 2 |
| *Linnemannia elongata* | AG-77 v.2 | 50 | 473 | 517,143 | 48 | 14,969 | C: 284  F: 5 |
| *Podila humilis* | PM1414 | 36 | 523 | 118,088 | 49 | 12,012 | C: 257  F: 6 |
| *Podilia verticillata ***** | NRRL6337 | 42 | 55 | 2,912,254 | 48 | 12,569 | C: 287  F: 3 |
| *Podila minutissima* | AD051 | 43 | 92 | 1,101,483 | 49 | 13,383 | C: 285  F: 3 |
| *Actinomortierella capitata* | AV005 | 36 | 47 | 2,013,012 | 50 | 10,395 | C: 286  F: 4 |
| *Basidiobolus meristosporus* | CBS 931.73 v1.0 | 89 | 1,366 | 106,019 | 43 | 16,111 | C: 281  F: 7 |

* The strain UK2014 was initially listed as *F. caledonius* but has been reidentified as *F. mosseae* (W. Wheeler personal communication) in line with phylogenomic analyses (49).

** The genus *Oehlia* is represented by *O. diaphana* strain DAOM227022. A genome assembly of this strain was previously included in phylogenomic analyses under the name *R. cerebriforme* (30, 54, 59), but based on molecular and morphological data, this strain was shown to represent *O. diaphana* (68).

*** DAOM229456 corresponds to MUCL43196 in (54) where this strain was published under the name *R. diaphanus but* phylogenetic analyses suggest that this strain actually belongs to *R. irregularis* (68).

**** Species name kept in this paper as previously earlier published, although the species name should probably be *P. humilis*.

# C: complete; F: fragmented

**Table S3.** Genes identified as Missing Glomeromycota Core Genes (MGCGs) by Tang et al. (2016), with summary of occurrence across genomes analyzed in this study. The column In Out indicates if the gene is present (P) in the outgroup *B. meristosporus*. In the Mucoromycota column, double asterix indicates that the gene family is not detected in the analyzed taxa within the mycorrhizal lineage Endogonales. Bold black font highlights 7 MGCGs that are missing only in Glomeromycota, bold green font indicate 6 genes missing in Glomeromycota and Endogonales, while grey font indicate 8 genes missing in all three phyla with 23 genomes assemblies in Glomeromycota (23 Glom), 13 in Mucoromycota (13 Muc) and 9 in Mortierellomycota (9 Mort).

| Pathway | Gene name | In  Out | Average nr. of gene copies | | | Nr. of genomes with copies | | |
| --- | --- | --- | --- | --- | --- | --- | --- | --- |
|  |  |  | Glom. | Muc. | Mort. | 23 Glom. | 13 Muc. | 9 Mort. |
| Fatty acid synthesis | **FAS1** | P | 0 | 1.2 | 1 | 0 | 12 | 9 |
|  | **FAS2** | P | 0 | 1.7 | 2 | 0 | 12 | 9 |
| Thiamine metabolism  /transport | **THI4** | P | 0 | 0.6 | 1 | 0 | 8** | 9 |
|  | **THI6** | P | 0 | 0.7 | 1 | 0 | 9** | 9 |
|  | THI7 | P | 0 | 0.2 | 0 | 0 | 3 | 9 |
|  | **THI20** | P | 0 | 0.8 | 1 | 0 | 10** | 9 |
|  | **THI21** | P | 0 | 0.8 | 1 | 0 | 10** | 9 |
|  | **THI22** | P | 0 | 0.8 | 1 | 0 | 10** | 9 |
|  | THI72 | P | 0 | 0.2 | 0 | 0 | 3 | 0 |
|  | NRT1 | P | 0 | 0.2 | 0 | 0 | 3 | 0 |
| Detoxifica-tion  /stress response | **RHR2** | P | 0 | 1.2 | 1.2 | 0 | 12 | 8 |
|  | **ARR3** | P | 0 | 1.2 | 1.3 | 0 | 11 | 8 |
|  | **DOG1** | P | 0 | 1.1 | 0.9 | 0 | 11 | 8 |
|  | **DOG2** | P | 0 | 1 | 1 | 0 | 11 | 9 |
|  | DSD1 | - | 0.04 | 0.9 | 0 | 1 | 12 | 0 |
|  | YHB1* | - | 0.04 | 0 | 0 | 1 | 0 | 0 |
|  | PUG1 | - | 0 | 0 | 0 | 0 | 0 | 0 |
|  | RTA1 | - | 0 | 0 | 0 | 0 | 0 | 0 |
| ER quality control | **PNG1** | - | 0 | 0.8 | 1 | 0 | 10 | 9 |
|  | MNN2 | - | 0 | 0 | 0 | 0 | 0 | 0 |
|  | MNN5 | - | 0 | 0 | 0 | 0 | 0 | 0 |
| Proteases  /peptidases | ECM14 | P | 0.04 | 0.3 | 5.6 | 1 | 2 | 9 |
| Sulfonate catabolism | **JLP1** | P | 0 | 1.8 | 2.2 | 0 | 10** | 9 |
| Sugar metabolism | SUC2 | - | 0 | 0.1 | 0 | 0 | 1 | 0 |
| Allantoin permease | DAL4 | P | 0 | 0.2 | 0 | 0 | 3 | 0 |
| Alcohol metabolism | ADH4 | P | 0.1 | 0.7 | 1 | 1 | 8 | 9 |
|  | AAD15 | P | 0 | 0.3 | 0 | 0 | 3 | 0 |
| Uracil metabolism | FUI1 | P | 0 | 0.2 | 0 | 0 | 3 | 0 |
|  | FUR4 | P | 0 | 0.2 | 0 | 0 | 3 | 0 |
| Chaperones | MCX1 | P | 0.04 | 1.5 | 1.4 | 1 | 12 | 9 |
| Aromatic ami-acid metabolism | ARO8 | - | 0.2 | 1.7 | 2.2 | 5 | 13 | 9 |
|  | ARO9 | - | 0.2 | 1.7 | 2.2 | 4 | 13 | 9 |
| Channels  /transporters | TOK1 | P | 0 | 0 | 0 | 0 | 0 | 0 |
|  | PHO89 | P | 1.1 | 1.1 | 3 | 12 | 8 | 9 |
|  | MCH2 | P | 0.1 | 8 | 5.6 | 3 | 12 | 9 |
| Cell cycle | AXL2 | - | 0 | 0 | 0 | 0 | 0 | 0 |
| Others | URE2 | P | 1.7 | 0.3 | 0 | 15 | 3 | 0 |
|  | FRE8 | - | 0 | 0 | 0 | 0 | 0 | 0 |
|  | YLR278C | - | 0 | 0 | 0 | 0 | 0 | 0 |

*Also detected in *R. irregularis* by Kobayashi *et al*. (2018)

**Table S4**. CAZyme genes families defined as plant cell wall degrading enzymes (PCWDEs) often found to be contracted in ECM fungal lineages in previous studies (reference number in main text in parenthesis), with summary of occurrence across genomes in the three phyla with 23 genomes assemblies in Glomeromycota (Glom.), 13 in Mucoromycota (Muc.) and 9 in Mortierellomycota (Mort.). The column In Out indicates if the gene is present (P) in the outgroup B. meristosporus. In the Mucoromycota column, an asterix indicates that the gene family is recorded in at least one of the analyzed taxa in the mycorrhizal lineage Endogonales. Bold font highlights gene families missing only in Glomeromycota while grey font indicates gene families that are absent in the outgroup and recorded in such low number that these genes are possibly absent across all analyzed taxa.

| Decay activity (Ref.) | CAZyme family | In Out | Ave. nr. of gene copies | | | Nr. of genomes with copies | | |
| --- | --- | --- | --- | --- | --- | --- | --- | --- |
|  |  |  | Glom. | Muc. | Mort. | Glom.^5^ | Muc. | Mort. |
| LPMO (14, 21, 27, 28) | AA9 ^1^ | - | 0.1 | 0 | 0 | 1 | 0 | 0 |
| Laccase (27) | AA1 | P | 10.6 | 4.9 | 3.9 | 22 | 13* | 9 |
| POD (21, 27) | AA2 ^2^ | P | 1.9 | 2.5 | 1.9 | 23 | 13* | 9 |
| Carbohydrate binding module (14, 28) | CBM1 | - | 0 | 0.2 | 0 | 0 | 2 | 0 |
| Carbohydrate esterases (21) | CE1 | **P** | **0** | **0.8** | **1** | **0** | **10*** | **9** |
|  | CE16 | P | 2.4 | 4.9 | 0.3 | 23 | 13* | 3 |
| Cellobiohydrolases Endoglucanases (14, 21, 28) | GH6 | - | 0 | 0.1 | 0 | 0 | 1 | 0 |
|  | GH7 | - | 0.04 | 0 | 0 | 1 | 1 | 0 |
|  | GH12 ^3^ | - | 0 | 0.2 | 0 | 0 | 3* | 0 |
| Glycoside hydrolase (21, 28) | GH5 ^4^ | P | 4.6 | 7.2 | 8.4 | 23 | 13 | 9 |
| Xylanases (21, 28) | GH10 | - | 0.1 | 0.2 | 0 | 1 | 3* | 0 |
| Pectin-acting enzymes (21, 28) | GH28 | - | 0.04 | 3.6 | 0.7 | 1 | 12* | 5 |
|  | GH43 | - | 0.04 | 0.6 | 0 | 1 | 5* | 0 |
| Endoglucanase (27, 28) | GH45 | - | 0 | 0.5 | 0 | 0 | 4 | 0 |
| Xylosidas (21) | GH3 | P | 0.04 | 5.1 | 0.8 | 1 | 13* | 7 |
| Polysaccharide lysase (14, 28) | PL1 | - | 0 | 0.2 | 0 | 0 | 2* | 0 |
|  | PL3 | - | 0 | 0 | 0.7 | 0 | 0 | 5 |

^1^ AA9 was previously classified as GM61

^2^ AA2 encompass peroxidases listed as POD (21)

^3^ GH12 was not included in (14)

^4^ In our analysis GH5 has not been divided into subgroups and the numbers are thus not directly comparable to earlier studies.

^5^ Note that gene families detected only once were all detected in *Acaulospora colombiana*

**Table S5**. CAZyme genes families identified in this study as missing only in Glomeromycota. Summary of occurrence across genomes in the three phyla 23 assemblies of Glom., 13 Mucoromycota (Muc.) and 9 Mortierellomycota (Mort.). The column In Out indicates if the gene is present (P) in the outgroup B. meristosporus. Note that CE1 is not listed in this table since its already presented in Table S4.

| Short information | CAZyme family | In  Out | Total nr. of gene copies | | | Nr. of genomes with copies | | |
| --- | --- | --- | --- | --- | --- | --- | --- | --- |
|  |  |  | Glom. | Muc. | Mort. | Glom. | Muc. | Mort. |
| Chitin-binding^1^ * | CBM5 | P | 0 | 0.3 | 1.2 | 0 | 2 | 8 |
|  | CBM12 | P | 0 | 0.3 | 0.3 | 0 | 2 | 3 |
| Mannosidase Galactosidase ^2^ | GH2 | - | 0 | 0.5 | 1.0 | 0 | 5 | 9 |
| Bacterial Glycoside hydrolases ^3^ | GH8 | P | 0 | 1.2 | 1.6 | 0 | 12 | 9 |
| Fucosidases ^4^ | GH29 | - | 0 | 1.2 | 0.1 | 0 | 8 | 1 |
| Fucosidases ^5^ | GH95 | - | 0 | 0.2 | 1.0 | 0 | 2 | 8 |
| Mainly phosphorylases ^6^ | GH65 | - | 0 | 0.2 | 0.9 | 0 | 2 | 7 |
| Various glucans ^7^ | GH74 | - | 0 | 0.3 | 0.1 | 0 | 3 | 1 |
| Acetylglucosaminidases ^8^ | GH89 | P | 0 | 0.2 | 2.2 | 0 | 2 | 9 |
| Mannanases ^9^ | GH134 | - | 0 | 1.5 | 0.1 | 0 | 5 | 1 |
| Alginate lyases ^10^ | PL14 | P | 0 | 2.9 | 2.7 | 0 | 13 | 9 |
| Mannuronan lyase ^11^** | PL36 | P | 0 | 1.9 | 0.1 | 0 | 12 | 1 |

1. <https://www.cazypedia.org/index.php/Carbohydrate_Binding_Module_Family_5>
2. <https://www.cazypedia.org/index.php/Glycoside_Hydrolase_Family_2>
3. <https://www.cazypedia.org/index.php/Glycoside_Hydrolase_Family_8>
4. <https://www.cazypedia.org/index.php/Glycoside_Hydrolase_Family_29>
5. <https://www.cazypedia.org/index.php/Glycoside_Hydrolase_Family_95>
6. <https://www.cazypedia.org/index.php/Glycoside_Hydrolase_Family_65>
7. <https://www.cazypedia.org/index.php/Glycoside_Hydrolase_Family_74>
8. <https://www.cazypedia.org/index.php/Glycoside_Hydrolase_Family_89>
9. <https://www.cazypedia.org/index.php/Glycoside_Hydrolase_Family_134>
10. <https://www.cazypedia.org/index.php/Polysaccharide_Lyase_Family_7>
11. Helbert et al 2019 <https://doi.org/10.1073/pnas.1815791116>

* Two related families of chitin binding modules, CBM5 so far only in bacteria

** This family has some similarity to PL14


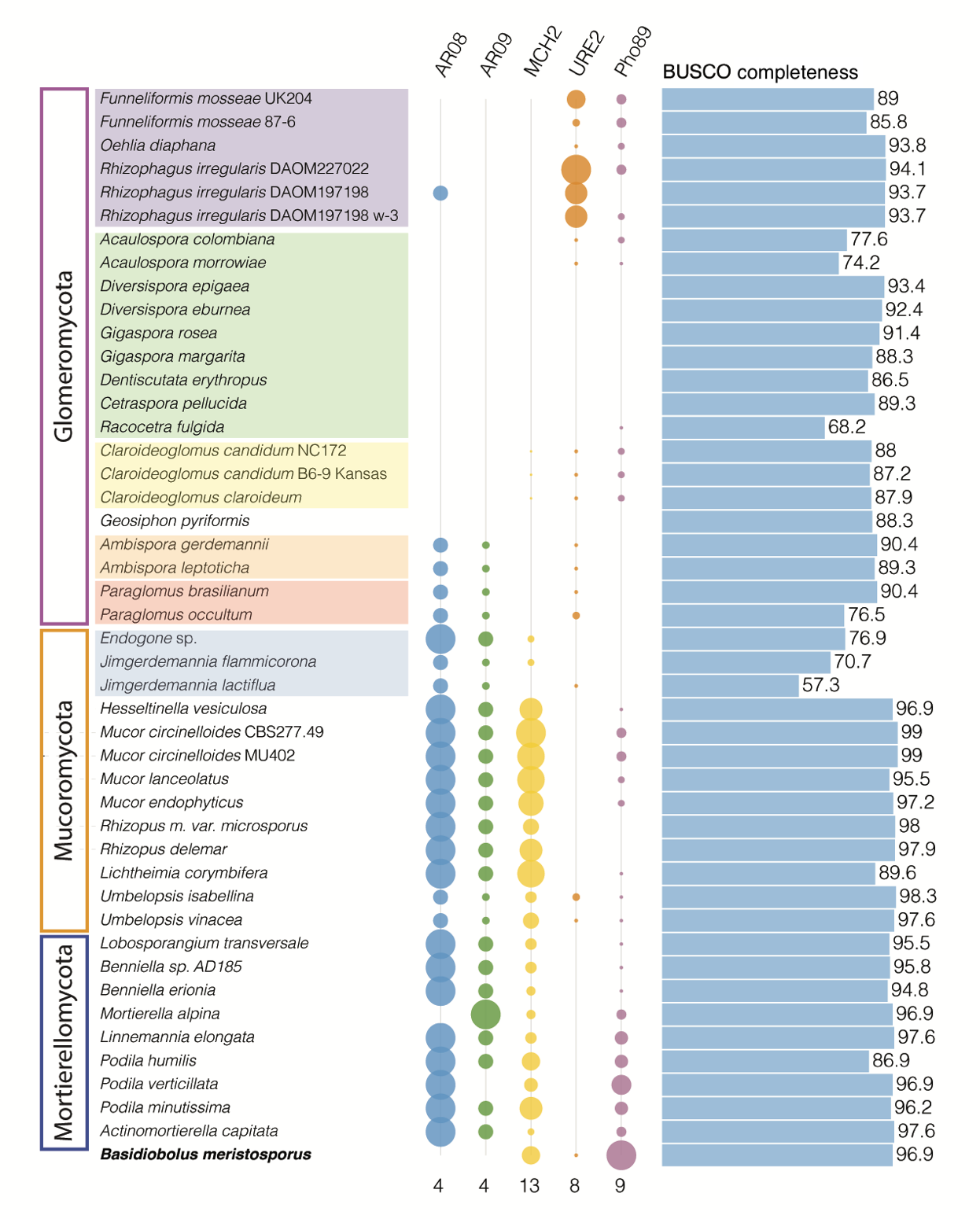


**Figure S1.** Gene copy number for five genes (AR08, AR09, MCH2, URE2, PHO89) previously identified as Missing Glomeromycota Core Genes (MGCGs) across taxa in the analyzed phyla. Circles represent the number of genes annotated in each assembly, scaled individually for each gene family for readability with maximum number of genes indicated at the bottom of each column. Estimated BUSCO completeness of the analyzed genome assemblies is indicated by bars to the right. Species are organized according to Fig. 1 and Endogonales as well as orders in Glomeromycota are highlighted by colored boxes. Observe that MCH2 is detected in one copy in each of the three Claroideoglomus genome assemblies.


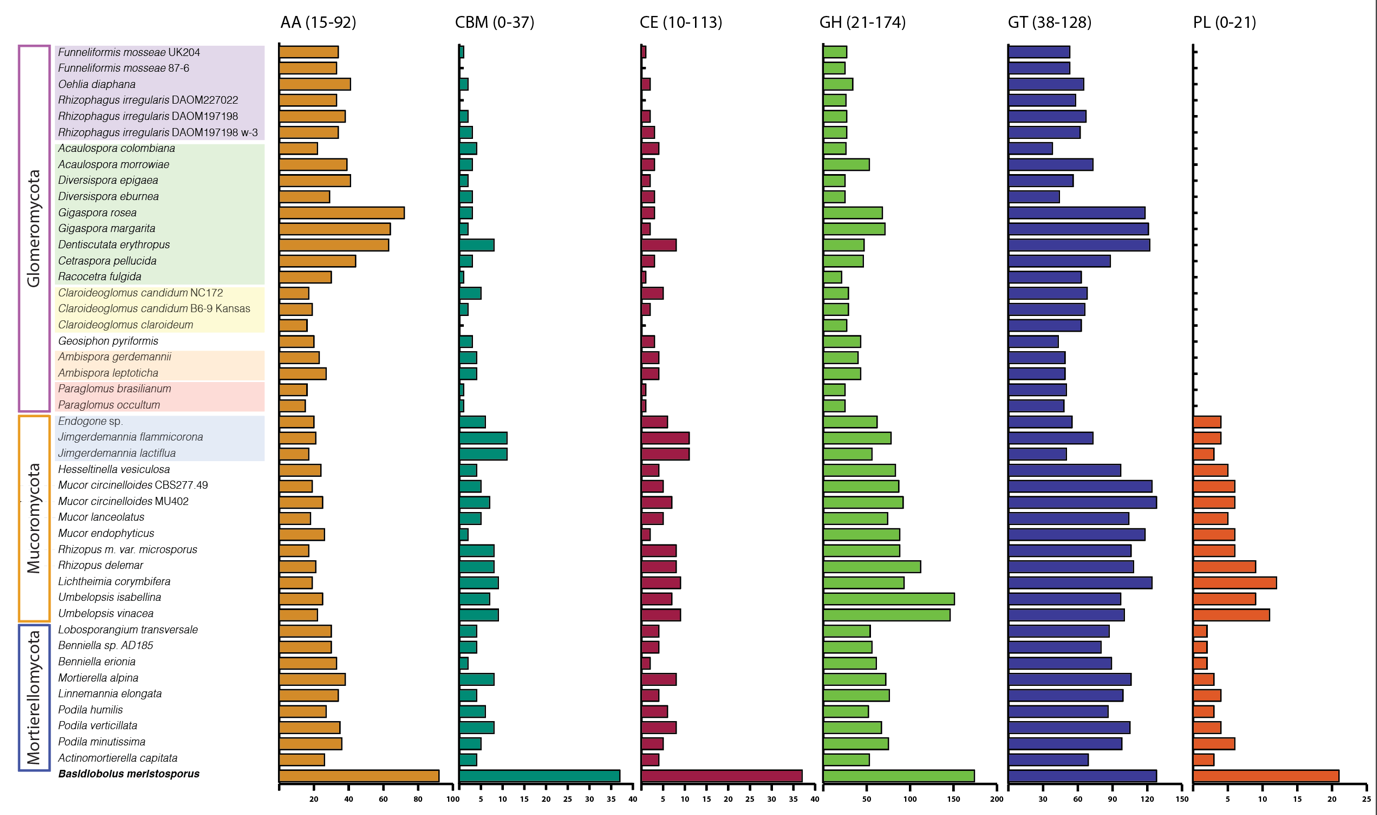


**Figure S2**. Total number of genes annotated into the six CAZyme classes: auxillary activity (AA) in okra, carbohydrate binding module (CBM) in dark green, carbohydrate esterase (CE) in maroon, glycoside hydrolase (GH) in green, glycosyl transferase (GT) in dark blue and polysaccharide lyase (PL) in red, across taxa in the analyzed phyla phylogeny. The range of genes in each class is given in parenthesis. Species are organized according to Fig. 1 and Endogonales as well as orders in Glomeromycota are highlighted by colored boxes.

**Figure S3**. Expansions (+) and contractions (-) of carbohydrate-active enzyme (CAZyme) gene families among Glomeromycota (purple), Mucoromycota (yellow) and Mortierellomycota (dark blue) as determined by computational analysis of gene family evolution (CAFE).

**Figure S4**. Expansions (red) and contractions (blue) of CAZyme family AA1 as determined by computational analysis of gene family evolution (CAFÉ).

**Figure S5**. Expansions (red) and contractions (blue) of CAZyme family AA3 as determined by computational analysis of gene family evolution (CAFÉ).

**Figure S6**. Expansions (red) and contractions (blue) of CAZyme family AA7 as determined by computational analysis of gene family evolution (CAFÉ).

**Figure S7**. Expansions (red) and contractions (blue) of CAZyme family AA11 as determined by computational analysis of gene family evolution (CAFÉ).

**Figure S8**. Expansions (red) and contractions (blue) of CAZyme family CE4 as determined by computational analysis of gene family evolution (CAFÉ).

**Figure S9**. Expansions (red) and contractions (blue) of CAZyme family CE10 as determined by computational analysis of gene family evolution (CAFÉ).

**Figure S10**. Expansions (red) and contractions (blue) of CAZyme family GH16 as determined by computational analysis of gene family evolution (CAFÉ).

**Figure S11**. Expansions (red) and contractions (blue) of CAZyme family GH47 as determined by computational analysis of gene family evolution (CAFÉ).

**Figure S12**. Expansions (red) and contractions (blue) of CAZyme family GT1 as determined by computational analysis of gene family evolution (CAFÉ).

**Figure S13**. Expansions (red) and contractions (blue) of CAZyme family GT2_chitin_synthetase as determined by computational analysis of gene family evolution (CAFÉ).

**Figure S14**. Expansions (red) and contractions (blue) of CAZyme family GT10 as determined by computational analysis of gene family evolution (CAFÉ).

**
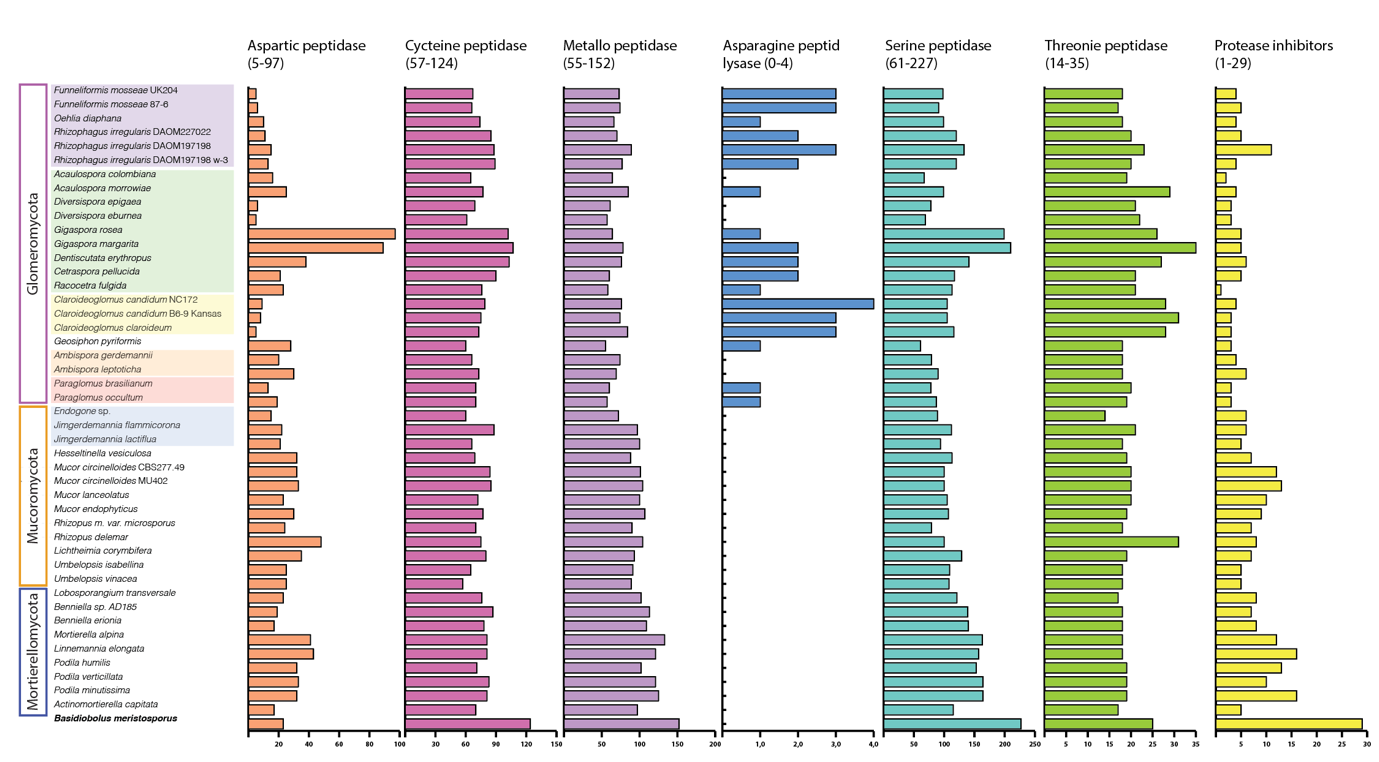
**

**Figure S15**. Total number of genes annotated into the eight families of proteolytic enzymes: aspartic peptidase (orange), cysteine peptidase (magenta), metallo peptidases (purple), asparagine peptide lysase (blue), serin peptidase (cyan), threonine peptidase (green) and protease inhibitors (yellow) across taxa in the analyzed phyla phylogeny. The range of genes in each class is given in parenthesis. Species are organized according to Fig. 1 and Endogonales as well as orders in Glomeromycota are highlighted by colored boxes.

**

**Figure S16**. Expansions (+) and contractions (-) of Peptidase gene families among Glomeromycota (purple), Mucoromycota (yellow) and Mortierellomycota (dark blue) as determined by computational analysis of gene family evolution (CAFE).

**Figure S17**. Expansions (red) and contractions (blue) of the peptidase family M13 as determined by computational analysis of gene family evolution (CAFE).

**Figure S18**. Expansions (red) and contractions (blue) of the peptidase family S09X as determined by computational analysis of gene family evolution (CAFE).

**Figure S19**. Expansions (red) and contractions (blue) of the peptidase family S12 as determined by computational analysis of gene family evolution (CAFE).

**Figure S20**. Expansions (red) and contractions (blue) of the peptidase family A01A as determined by computational analysis of gene family evolution (CAFE).

**Figure S21**. Expansions (red) and contractions (blue) of the peptidase family C19 as determined by computational analysis of gene family evolution (CAFE).

**Figure S22**. Expansions (red) and contractions (blue) of the peptidase family S09C as determined by computational analysis of gene family evolution (CAFE).

**Figure S23**. Expansions (red) and contractions (blue) of the peptidase family S10 as determined by computational analysis of gene family evolution (CAFE).
